# Efficacy and secondary infection risk of tocilizumab, sarilumab and anakinra in COVID‐19 patients: A systematic review and meta‐analysis

**DOI:** 10.1002/rmv.2295

**Published:** 2021-09-24

**Authors:** Jingwen Peng, Meihua Fu, Huan Mei, Hailin Zheng, Guanzhao Liang, Xiaodong She, Qiong Wang, Weida Liu

**Affiliations:** ^1^ Department of Medical Mycology Institute of Dermatology, Chinese Academy of Medical Science and Peking Union Medical College Nanjing Jiangsu China; ^2^ Jiangsu Key Laboratory of Molecular Biology for Skin Diseases and STIs Institute of Dermatology, Chinese Academy of Medical Science and Peking Union Medical College Nanjing Jiangsu China; ^3^ Center for Global Health School of Public Health, Nanjing Medical University Nanjing China

**Keywords:** anakinra, COVID‐19, meta‐analysis, sarilumab, tocilizumab

## Abstract

As the pandemic progresses, the pathophysiology of coronavirus disease 2019 (COVID‐19) is becoming clearer and the potential for immunotherapy is increasing. However, clinical efficacy and safety of immunosuppressants (including tocilizumab, sarilumab and anakinra) treatment in COVID‐19 patients are not yet known. We searched PubMed, Embase Medline, Web of Science and MedRxiv using specific search terms in studies published from 1 January 2020 to 20 December 2020. In total, 33 studies, including 3073 cases and 6502 controls, were selected for meta‐analysis. We found that immunosuppressant therapy significantly decreased mortality in COVID‐19 patients on overall analysis (odds ratio = 0.71, 95% confidence interval = 0.57–0.89, *p* = 0.004). We also found that tocilizumab and anakinra significantly decreased mortality in patients without any increased risk of secondary infection. In addition, we found similar results in several subgroups. However, we found that tocilizumab therapy significantly increased the risk of fungal co‐infections in COVID‐19 patients. This represents the only systematic review and meta‐analysis to investigate the efficacy and secondary infection risk of immunosuppressant treatment in COVID‐19 patients. Overall, immunosuppressants significantly decreased mortality but had no effect on increased risk of secondary infections. Our analysis of tocilizumab therapy showed a significantly increased risk of fungal co‐infections in these patients.

**Abbreviations**
ARDS: acute respiratory distress syndrome
CAR‐T: chimerical antigen receptor T cell
CI: confidence interval
COVID‐19: coronavirus disease 2019
CRS: cytokine release syndrome
ICU: intensive care unit
IDSA: Infectious Diseases Society of America
IL: interleukin
MAS: macrophage activation syndrome
MERS‐CoV: Middle East respiratory syndrome coronavirus
NLRP3: Nod‐like receptor protein‐3
OR: odds ratio
ORF3: open reading frame 3
SARS‐CoV‐2: severe acute respiratory syndrome coronavirus 2
sHLH: secondary hemophagocytic lymphocytosis
WHO: World Health Organization

## 1 INTRODUCTION

Severe acute respiratory syndrome coronavirus 2 (SARS‐CoV‐2) is a novel human pathogen, responsible for the largest ever global challenge to public health and humanity.^1^, ^2^, ^3^ Coronavirus disease 2019 (COVID‐19) is associated with a dysregulated immune response and hyperinflammation, which can lead to or exacerbate acute respiratory distress syndrome (ARDS) and multiorgan failure.^4^ As the pandemic progresses, the pathophysiology of COVID‐19 is becoming clearer and the potential of immunotherapy has increased.^4^ However, clinical efficacy and safety of immunosuppressants (including tocilizumab, sarilumab and anakinra) treatment in COVID‐19 patients are not yet known.

Severe acute respiratory syndrome coronavirus 2 infection results in a wide spectrum of clinical manifestations, ranging from asymptomatic or paucisymptomatic forms (with fever, dry cough, myalgia and malaise) to full‐blown viral pneumonia, which can lead to ARDS, multiorgan failure and death.^5^ Infection causes destruction of alveolar epithelial cells, activation of the innate immune system and dysregulation of adaptive immune responses, including the release of proinflammatory cytokines and chemokines.^6^, ^7^ The serum cytokine profile of some patients with moderate to severe COVID‐19 overlaps with those having macrophage activation syndrome (MAS) and secondary hemophagocytic lymphocytosis (sHLH). Macrophage activation syndrome is characterised by excessive inflammation, fever, elevated ferritin levels, lung involvement and a range of symptoms including sHLH. Viruses are known as MAS/sHLH triggers, and ferritin levels are associated with MAS and mortality in COVID‐19 patients.^8^, ^9^ Endogenous interleukin 1 (IL‐1) is a pro‐inflammatory cytokine that induces interleukin 6 (IL‐6) production in monocytes and macrophages and is elevated in COVID‐19 disease, MAS and other conditions such as severe chimerical antigen receptor T cell (CAR‐T)‐mediated cytokine release syndrome (CRS).^4^ Moreover, plasma IL‐6 levels are elevated in intensive care unit (ICU) patients with COVID‐19 and appear to be positively correlated with mortality.^10^ Since the clinical severity of COVID‐19 appears to be related to a cytokines storms, with an overproduction of soluble inflammatory mediators, several immunosuppressants agents are currently under investigation, but results thus far are inconclusive.

However, it is essential to perform well‐controlled clinical trials to confirm the efficacy of immunosuppressants to provide data for evidence‐based decision‐making. Therefore, amidst the lack of robust evidence regarding the use of immunosuppressants treatment for COVID‐19, we aimed to explore the efficacy and safety of immunosuppressants therapy in a systematic review and meta‐analysis involving 33 studies in 3073 cases with COVID‐19 and 6502 controls. We hope that this will help to inform clinical management of the secondary infection.

## 2 METHODS

### 2.1 Study selection

**FIGURE 1:**
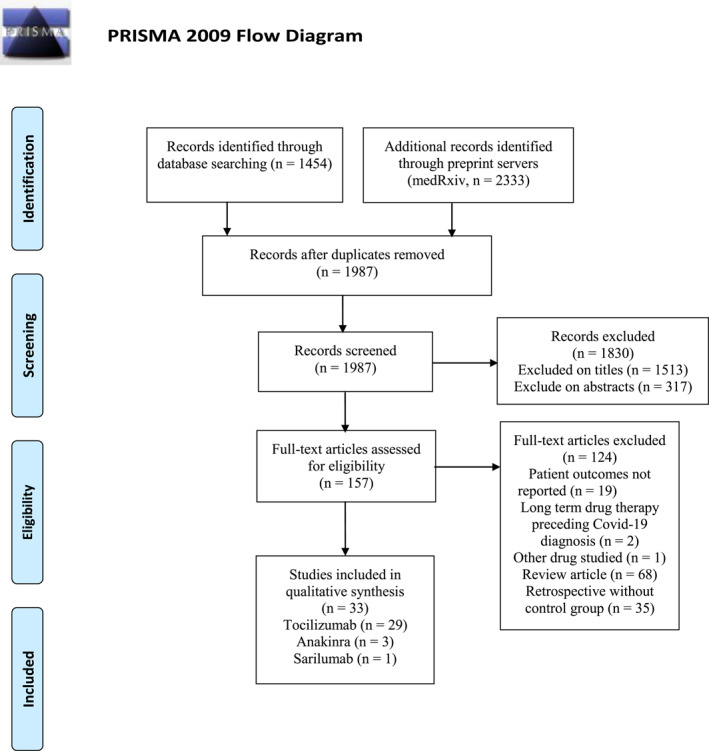
Flow diagram of the study selection process. *Source*: Moher D, Liberati A, Tetzlaff J, Altman DG, The PRISMA Group. Preferred reporting items for systematic reviews and meta‐analyses: the PRISMA statement. *PLoS Med*. 2009;6(6):e1000097. doi:10.1371/journal.pmed1000097. For more information, visit www.prisma‐statement.org

The Preferred Reporting Items for Systematic Reviews and Meta‐Analyses (PRISMA) Checklist was used to improve the reporting of our meta‐analysis. We searched PubMed, EMBASE, MEDLINE, Web of Science and MedRxiv using the search terms immunosuppressants, anakinra, sarilumab, siltuximab, tocilizumab, bacterial/fungal co‐infection, coronavirus, severe acute respiratory syndrome coronavirus 2, SARS‐CoV‐2, 2019‐nCoV and COVID‐19 for studies published from 1 January 2020 to 20 December 2020, and we manually searched the references of select articles for additional relevant articles (Figure 1).

### 2.2 Data extraction and verification

The inclusion criteria for the meta‐analysis were as follows: (1) research focus on immunosuppressants (such as tocilizumab, anakinra, sarilumab, siltuximab, sirukumab, etc.) and COVID‐19; (2) the number of cases and controls; (3) randomised controlled trial (RCT) or retrospective study, including case‐control study and cohort study; (4) papers where the full text was available; (5) including mortality and the number of secondary infection; and (6) studies on human beings. The exclusion criteria of the meta‐analysis were as follows: (1) review; (2) case report; (3) animal or cell study; (4) data are incomplete, and outcome effects are unclear; and (5) repeat the report.

Information pertaining to the enrolled studies is listed in Table 1, including: (I) the author's name, (II) country or region of origin, (III) ethnicity or race, (IV) severity, (V) dose, (VI) study design, (VII) journal, (VIII) number of case, (IX) number of controls, (X) number of deaths in the case, (XI) number of deaths in the controls, (XII) number of secondary infections in the case, (XIII) number of secondary infection in the controls, and (XIV) drug. Firstly, three authors (Jingwen Peng, Huan Mei and Meihua Fu) independently screened the citations that met our inclusion criteria and extracted all data. If at least two of the three researchers agreed, the study was included in the meta‐analysis. Next, everyone extracted while the other cross‐checked the data. Disagreements were resolved by reviewing and discussing.

**TABLE 1:** Characteristics of all studies describing mortality and secondary infection risk in the meta‐analysis

Author	Country	Race	Disease severity	Dose (mg)	Study type	Journal	Case size	Case	Control	Death		Secondary infection (fungal)		Drug
JH Stone	USA	Mix	Mix	8 mg/kg	RCT	Publish	>100	161	82	9	3	13	14	Tocilizumab
Campochiaro	Italy	Caucasian	Severe	400	Case‐control	Publish	<100	32	33	5	11	4	4	Tocilizumab
Capra	Italy	Caucasian	Moderate	400–800	Case‐control	Publish	<100	62	23	2	11	0	0	Tocilizumab
Guaraldi	Italy	Caucasian	Severe	8 mg/kg	Multicentre case‐control	Publish	>100	179	365	13	73	24 (6)	14 (2)	Tocilizumab
Kewan	USA	Mix	Severe	8 mg/kg	Case‐control	Publish	<100	28	23	3	2	5 (1)	5 (2)	Tocilizumab
Quartuccio	Italy	Caucasian	Severe	<400	Case‐control	Publish	<100	42	69	7	0	17	0	Tocilizumab
Ip	USA	Mix	Critical	400	Multicentre case‐control	Publish	>100	134	413	62	231	18	44	Tocilizumab
Kimmig	USA	Mix	Critical	400	Case‐control	Publish	<100	54	57	19	11	29 (3)	16 (0)	Tocilizumab
Somers	USA	Mix	Severe	8 mg/kg	Case‐control	Publish	<100	78	76	7	20	42 (3)	20 (2)	Tocilizumab
Potere	Italy	Caucasian	Severe	<400	Case‐control	Publish	<100	40	40	2	11	1	3	Tocilizumab
Hermine	France	Caucasian	Mix	8 mg/kg	RCT	Publish	<100	63	67	7	8	2 (0)	11 (2)	Tocilizumab
Ruiz‐Antorán	Spain	Caucasian	Severe	400–800	Multicentre case‐control	Publish	>100	268	238	45	75	124	72	Tocilizumab
Hill	USA	Mix	Severe	400	Case‐control	Publish	<100	43	45	9	15	4	2	Tocilizumab
Tsai	USA	Mix	Severe	400–800	Case‐control	Publish	<100	66	66	18	18	4	4	Tocilizumab
Albertini	France	Caucasian	Severe	400–800	Case‐control	Publish	<100	22	22	3	2	0	0	Tocilizumab
Salvarani	Italy	Caucasian	Severe	8 mg/kg	RCT	Publish	<100	60	66	2	1	1	4	Tocilizumab
Canziani	Italy	Caucasian	Severe	8 mg/kg	Case‐control	Publish	<100	64	64	17	24	20	25	Tocilizumab
De Rossi	Italy	Caucasian	Severe	400	Case‐control	Publish	<100	90	68	7	34	6	4	Tocilizumab
Eimer	Sweden	Caucasian	Severe	8 mg/kg	Case‐control	Publish	<100	29	58	10	26	9	20	Tocilizumab
Galvan‐Roman	Spain	Caucasian	Severe	8 mg/kg	Case‐control	Publish	<100	58	88	14	16	3	7	Tocilizumab
Pettit	USA	Mix	NA	400	Case‐control	Publish	<100	74	74	29	17	17 (2)	6 (1)	Tocilizumab
Potere	Italy	Caucasian	Moderate	<400	Case‐control	Publish	<100	10	10	0	0	0	0	Tocilizumab
Rodríguez‐Bano	Spain	Caucasian	NA	400–800	Case‐control	Publish	<100	88	339	2	41	11	36	Tocilizumab
Rojas‐Marte	USA	Mix	Severe	NA	Case‐control	Publish	<100	96	97	43	55	16 (4)	26 (3)	Tocilizumab
Salama	USA	Mix	NA	8 mg/kg	RCT	Publish	>100	249	128	26	11	25	16	Tocilizumab
Gupta	USA	Mix	Critical	NA	Multicentre case‐control	Publish	>100	433	3491	125	1419	29	285	Tocilizumab
Zheng	China	Asian	Mix	400–800	Case‐control	Publish	<100	92	89	9	1	0	0	Tocilizumab
Rosas	USA	Mix	Severe	8 mg/kg	RCT	medRxiv	>100	294	144	58	28	113	58	Tocilizumab
Carvalho	Brazil	Caucasian	Critical	400	Case‐control	medRxiv	<100	29	24	5	4	11 (6)	4 (1)	Tocilizumab
Della‐Torre	Italy	Caucasian	Severe	400	Case‐control	Publish	<100	28	28	2	5	6	5	Sarilumab
Kooistra	Netherlands	Caucasian	Critical	<400	Case‐control	Publish	<100	21	39	4	7	7	9	Anakinra
Cauchois	France	Caucasian	Severe	<400	Case‐control	Publish	<100	12	10	0	1	0	0	Anakinra
Huet	France	Caucasian	Severe	<400	Case‐control	Publish	<100	52	44	7	22	0	0	Anakinra

*Note*: Mix of severity, symptoms of the disease include moderate, severe and critical; mix of race, including, Asian, Caucasian, African and so on.

Abbreviations: NA, no appearance; RCT, randomised controlled trial.

### 2.3 Statistical analysis

The statistical significance of the pooled odds ratio (OR) was determined with the *Z*‐test, considering the values of *p *< 0.05 to be statistically significant. Data were pooled from the meta‐analysis with the random‐effects model using the DerSimonian and Laird method and the fixed‐effects model using the Mantel‐Haenszel method. In cases where *I* ^2^ < 50% and the *p*‐value for heterogeneity was >0.10, thus indicating an absence of heterogeneity between studies being compared, the fixed‐effects model was used to evaluate the summary ORs. Conversely, if *I* ^2^ ≥ 50% or the *p*‐value for heterogeneity was ≤0.10, thus indicating a higher degree of heterogeneity between studies but still met our inclusion criteria for meta‐analysis, we used the random‐effects model to evaluate the summary ORs. To evaluate the influence of individual data sets on overall pooled ORs, we conducted forest plot analysis to determine the stability of our results. We also carried out sensitivity analysis in which a single study within the overall meta‐analysis was deleted one at a time. We applied Funnel plots and Egger's linear regression test to assess publication bias. All statistical analyses were carried out using STATA version 11.0 (Stata Corporation College Station).

## 3 RESULTS

### 3.1 Study selection and characteristics

The combined search terms yielded all related articles, and the primary review of titles and abstracts identified 157 articles that warranted a full manuscript review. Through literature searches and selection based on inclusion criteria, 33 articles were identified after reviewing potentially relevant articles (Figure 1). In total, 33 studies, including 3073 cases and 6502 controls, met the inclusion criteria and were selected for meta‐analysis. Eight articles reported that 38 patients had a fungal co‐infection, with an overall fungal co‐infection rate of 2.75%. The characteristics of the selected studies are summarised in Table 1.

### 3.2 Mortality

**FIGURE 2:**
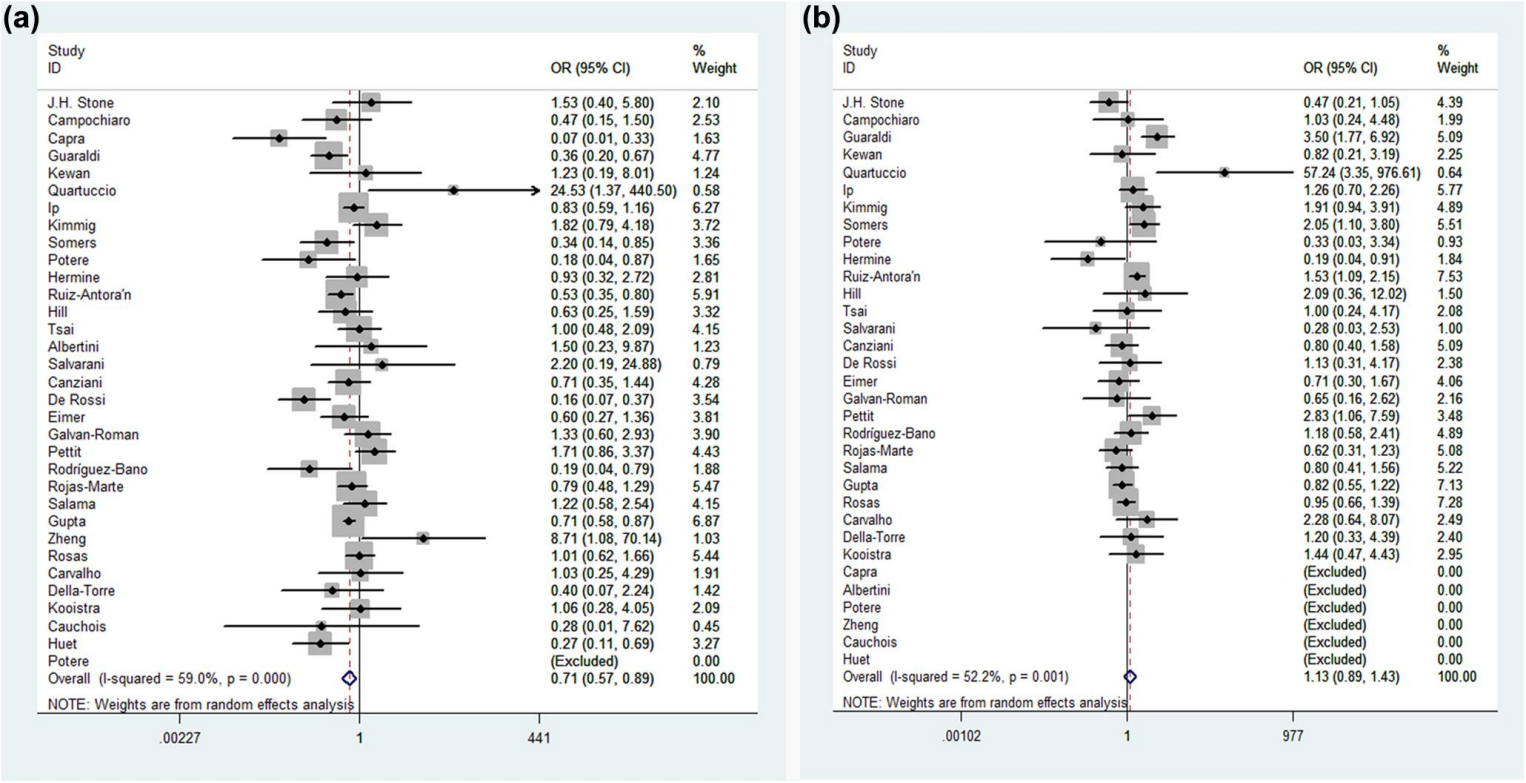
Forest plot of the associations between immunosuppressants and mortality and secondary infection risk in COVID‐19 patients. Forest plot of association between (a) immunosuppressants and mortality and (b) immunosuppressants and secondary infection risk

We performed a meta‐analysis of 33 observational studies (3073 cases and 6502 controls) which reported significantly decreased mortality after patients received immunosuppressants in the overall analysis (OR = 0.71, 95% confidence interval [CI] = 0.57–0.89, *p* = 0.004). When using subgroup analysis, we found significant associations within the mixed race subgroup (OR = 0.82, 95% CI = 0.72–0.95, *p* = 0.006), Caucasian subgroup (OR = 0.51, 95% CI = 0.35–0.74, *p* = 0.000), moderate subgroup (OR = 0.07, 95% CI = 0.01–0.33, *p* = 0.001), severe subgroup (OR = 0.06, 95% CI = 0.45–0.80, *p* = 0.001), critical (OR = 0.78, 95% CI = 0.65–0.92, *p* = 0.003), dose of 8 mg/kg subgroup (OR = 0.76, 95% CI = 0.60–0.95, *p* = 0.019), no specific dose subgroup (OR = 0.72, 95% CI = 0.60–0.87, *p* = 0.001), case‐control subgroup (OR = 0.67, 95% CI = 0.46–0.97, *p* = 0.035), multicentre case‐control subgroup (OR = 0.63, 95% CI = 0.48–0.83, *p* = 0.001), journal type subgroup (OR = 0.69, 95% CI = 0.54–0.88, *p* = 0.003), case size of >100 subgroup (OR = 0.73, 95% CI = 0.57–0.94, *p* = 0.015), case size of <100 subgroup (OR = 0.69, 95% CI = 0.48–0.99, *p* = 0.042), tocilizumab subgroup (OR = 0.74, 95% CI = 0.58–0.94, *p* = 0.013) and *anakinra* subgroup (OR = 0.41, 95% CI = 0.19–0.85, *p* = 0.017), but not the remaining subgroups. Interestingly, when compared to the standard treatment group, we found increased mortality after COVID‐9 patients had received immunosuppressants in the Asian subgroup analysis (OR = 8.71, 95% CI = 1.08–10.14, *p* = 0.042). Specific data are summarised in Figure 2a and Table 2.

**TABLE 2:** Mortality and secondary infection risk after COVID‐19 patients received immunosuppressants therapy

Mortality	OR (95% CI)	*P*	*P*_*h*_	*I* ^2^%	*P*_*b*_
Total	**0.71 (0.57–0.89)**	**0.004**	0.000	59.0	0.946
Race					
Mix	**0.82 (0.72–0.95)**	**0.006**	0.114	34.5	‐
Asian	**8.71 (1.08–10.14)**	**0.042**	‐	‐	‐
Caucasian	**0.51 (0.35–0.74)**	**0.000**	0.002	54.6	‐
Severity					
Mix	1.76 (0.86–3.64)	0.124	0.160	45.4	‐
Severe	**0.06 (0.45–0.80)**	**0.001**	0.004	51.9	‐
Critical	**0.78 (0.65–0.92)**	**0.003**	0.262	23.9	‐
NA	0.88 (0.32–2.44)	0.810	0.019	74.7	‐
Moderate	**0.07 (0.01–0.33)**	**0.001**	‐	‐	‐
Dose					
8 mg/kg	**0.76 (0.60–0.95)**	**0.019**	0.106	36.7	‐
400 mg	0.73 (0.42–1.27)	0.260	0.001	70.9	‐
400–800 mg	0.59 (0.25–1.42)	0.241	0.002	73.9	‐
<400 mg	0.62 (0.16–2.37)	0.489	0.016	67.3	‐
NA	**0.72 (0.60–0.87)**	**0.001**	0.695	0.0	‐
Study type					
RCT	1.11 (0.77–1.59)	0.588	0.937	0.0	‐
Case‐control	**0.67 (0.46–0.97)**	**0.035**	0.000	64.5	‐
Multicentre case‐control	**0.63 (0.48–0.83)**	**0.001**	0.080	55.7	‐
Journal					
Publish	**0.69 (0.54–0.88)**	**0.003**	0.000	60.4	‐
MedRxiv	1.02 (0.64–1.62)	0.944	0.980	0.0	‐
Case size					
>100	**0.73 (0.57–0.94)**	**0.015**	0.052	52.0	‐
<100	**0.69 (0.48–0.99)**	**0.042**	0.000	61.9	‐
Drug					
Tocilizumab	**0.74 (0.58–0.94)**	**0.013**	0.000	61.5	‐
Sarilumab	0.40 (0.07–2.24)	0.297	‐	‐	‐
Anakinra	**0.41 (0.19–0.85)**	**0.017**	0.251	27.6	‐

Secondary infection risk	OR (95% CI)	*P*	*P*_*h*_	*I* ^2^%	*P*_*b*_
Total	1.13 (0.89–1.43)	0.309	0.001	52.2	0.914
Race					
Mix	1.07 (0.81–1.41)	0.637	0.038	46.5	‐
Caucasian	1.17 (0.79–1.74)	0.439	0.006	54.5	‐
Severity					
Mix	**0.37 (0.18–0.74)**	**0.005**	0.308	3.7	‐
Severe	1.17 (0.85–1.61)	0.343	0.006	52.7	‐
Critical	1.12 (0.85–1.46)	0.426	0.191	34.5	‐
NA	1.22 (0.79–1.87)	0.372	0.114	54.0	
Dose					
8 mg/kg	0.91 (0.59–1.38)	0.647	0.001	65.5	‐
400 mg	**1.62 (1.15–2.26)**	**0.005**	0.849	0.0	‐
400–800 mg	**1.44 (1.07–1.93)**	**0.017**	0.713	0.0	‐
<400 mg	2.56 (0.19–34.51)	0.478	0.008	79.5	‐
NA	0.77 (0.54–1.08)	0.132	0.491	0.0	‐
Study type					
RCT	*0.76 (0.57–1.01)*	*0.057*	0.157	39.6	‐
Case‐control	1.24 (0.91–1.68)	0.168	0.090	32.5	‐
Multicentre case‐control	1.46 (0.87–2.45)	0.147	0.003	78.8	‐
Journal					
Publish	1.12 (0.86–1.45)	0.390	0.001	54.2	‐
MedRxiv	1.03 (0.72–1.47)	0.871	0.197	40.0	‐
Case size					
>100	1.12 (0.77–1.62)	0.555	0.001	73.4	‐
<100	1.14 (0.82–1.57)	0.435	0.031	40.6	‐
Drug					
Tocilizumab	1.12 (0.87–1.43)	0.376	0.000	55.8	‐
Sarilumab	1.20 (0.33–4.39)	0.783	‐	‐	‐
Anakinra	1.44 (0.47–4.43)	0.520	‐	‐	‐

Fungal co‐infection	OR (95%CI)	*P*	*P*_*h*_	*I* ^2^%	*P*_*b*_
Total	**2.02 (1.05–3.90)**	**0.036**	0.400	3.8	0.501
Race					
Caucasian	**2.86 (1.03–7.90)**	**0.043**	0.144	48.5	‐
Mix	1.56 (0.65–3.71)	0.318	0.690	0.0	‐
Severity					
Severe	1.89 (0.82–4.35)	0.134	0.286	20.8	‐
Critical	*5.76 (0.99–33.45)*	*0.051*	0.833	0.0	‐
NA	2.00 (0.18–22.54)	0.575	‐	‐	‐
Dose					
8 mg/kg	1.58 (0.65–3.84)	0.315	0.137	45.7	‐
400 mg	**4.23 (1.05–17.08)**	**0.043**	0.770	0.0	‐
NA	1.35 (0.29–6.18)	0.701	‐	‐	‐
Study type					
RCT	0.21 (0.01–4.51)	0.321	‐	‐	‐
Case‐control	1.91 (0.86–4.22)	0.109	0.653	0.0	‐
Multicentre case‐control	**6.12 (1.22–30.61)**	**0.027**	‐	‐	‐
Journal					
Publish	**1.78 (0.89–3.59)**	**0.104**	0.366	8.1	‐
MedRxiv	4.97 (0.56–44.15)	0.151	‐	‐	‐
Case size					
>100	**6.12 (1.22–30.61)**	**0.027**	‐	‐	‐
<100	1.57 (0.75–3.26)	0.230	0.548	0.0	‐

*Notes*: Mix of race, including, Asian, Caucasian, African and so on; mix of severity, symptoms of the disease include moderate, severe and critical; *P*_h_, *p* value of heterogeneity, *p* value of *Q*‐test for the heterogeneity test; *I* ^2^, 0–25, no heterogeneity; 25–50, modest heterogeneity; 50, high heterogeneity. Bold values indicates statistically significant results.

Abbreviations: CI, confidence interval; COVID‐19, coronavirus disease 2019; NA, no appearance; OR, odds ratio; RCT, randomised controlled trial.

### 3.3 Secondary infection risk

No significant associations were observed between immunosuppressants and an elevated risk of secondary infection in the overall analysis (OR = 1.13, 95% CI = 0.89‐1.43, *p* = 0.309). We did, however, find significantly decreased secondary infection risk after COVID‐19 patients received immunosuppressants in the mixed‐severity subgroup (OR = 0.37, 95% CI = 0.18–0.74, *p* = 0.005). However, we found significantly increased secondary infection risk after COVID‐19 patients received immunosuppressants in the dose of 400‐mg subgroup (OR = 1.62, 95% CI = 1.15–2.26, *p* = 0.005) and dose of 400 to 800‐mg subgroup (OR = 1.44, 95% CI = 1.07–193, *p* = 0.017). In addition, an edge effect may exist in the RCT subgroup (OR = 0.76, 95% CI = 0.57–1.01, *p* = 0.057). Data on the association between risks of immunosuppressants and secondary infection are summarised in Table 2 and Figure 2b.

### 3.4 Fungal co‐infection risk

**FIGURE 3:**
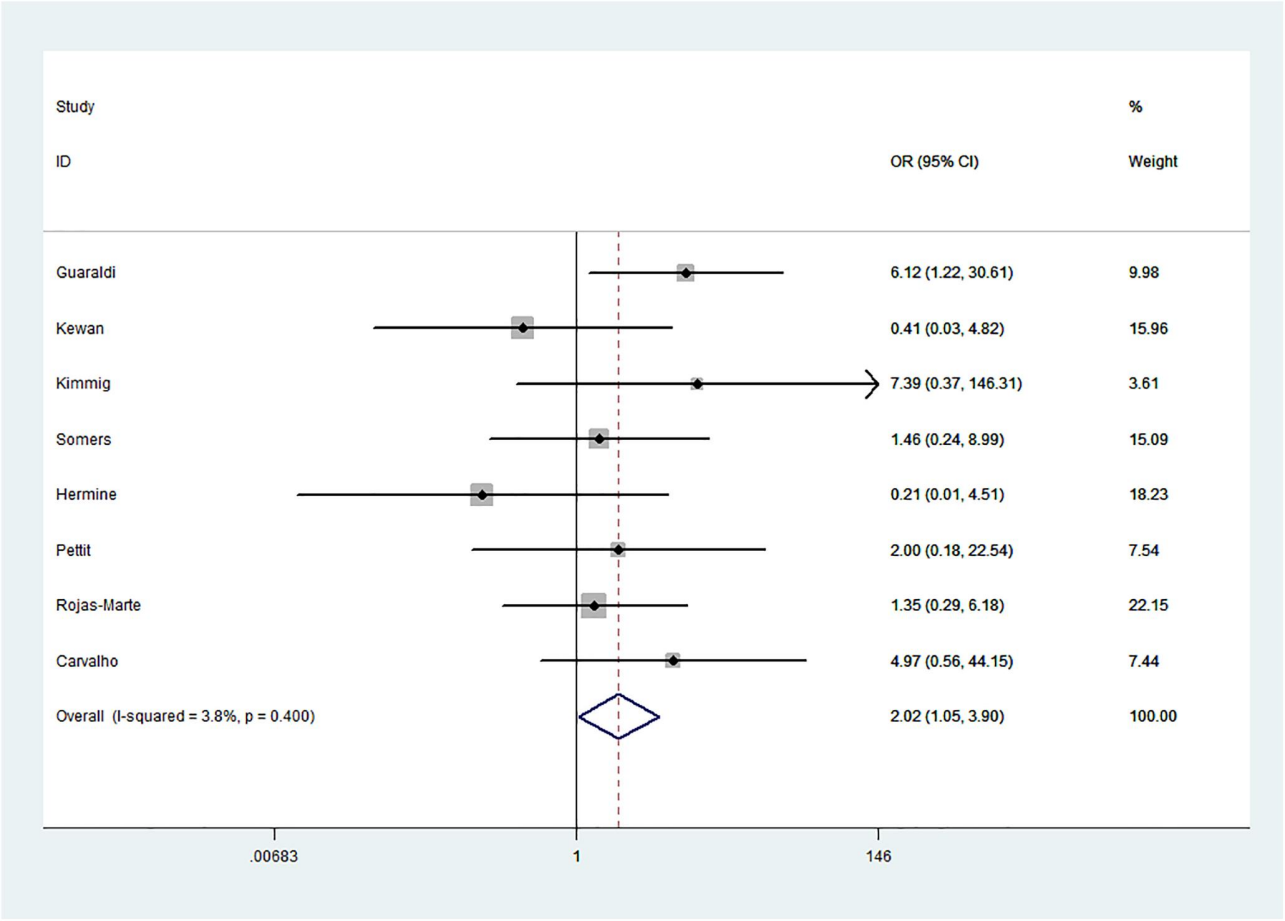
Forest plot of tocilizumab therapy and fungal co‐infection risk in COVID‐19 patients

We found tocilizumab therapy significantly increased the risk of fungal co‐infections in COVID‐19 patients, eight observational studies (601 cases and 783 controls), in the overall analysis (OR = 2.02, 95% CI = 1.05–3.90, *p* = 0.036). In subgroup analysis, we also revealed a significantly increased risk of fungal co‐infection after COVID‐19 patients received tocilizumab in the Caucasian subgroup (OR = 2.86, 95% CI = 1.03–7.90, *p* = 0.043), in the dose of 400‐mg subgroup (OR = 4.23, 95% CI = 1.05–17.08, *p* = 0.043), multicentre case‐control subgroup (OR = 6.12, 95% CI = 1.22–30.61, *p* = 0.027) and case size of >100 subgroup (OR = 6.12, 95% CI = 1.22–30.61, *p* = 0.027). However, we also found an edge effect in the critical subgroup (OR = 5.76, 95% CI = 0.99–33.45, *p* = 0.051). Specific data are summarised in Table 2 and Figure 3.

### 3.5 Publication bias and sensitivity analysis

Begg's funnel plot and Egger's test were performed to assess publication bias. We additionally conducted sensitivity analyses by omitting one study at a time in the calculation of a summary outcome. Although the sample sizes for cases in all eligible studies varied, corresponding pooled proportions and 95% CIs were not qualitatively altered between studies with small and large sample sizes. No other single study influenced pooled proportion and 95% CI qualitatively.

## 4 DISCUSSION

The World Health Organization has declared that COVID‐19 may progress to a pandemic associated with substantial morbidity and mortality and is a public health emergency of international concern as of 1 February 2020.^11^, ^12^, ^13^, ^14^ At the time this manuscript was compiled, over 82 million laboratory‐diagnosed cases of COVID‐19 had been reported spanning 212 countries or regions and contributing to over 1,700,000 deaths.

The proinflammatory cytokine IL‐1β is activated and secreted upon initiation of the inflammasome. Nod‐like receptor protein‐3, the most‐frequently studied inflammasome, is activated by danger signals and speculated to be viroporin A, E protein or open reading frame 3 proteins from SARS‐CoV and MERS‐CoV. Anakinra, a recombinant IL‐1 receptor a antagonist, has proven effective in treating sHLH, and it is currently used in IL‐1‐induced autoinflammatory diseases.^4^

In a recent study, *anakinra* significantly reduced both the need for invasive mechanical ventilation in the ICU and mortality among patients with severe COVID‐19, with no serious side‐effects.^6^, ^15^, ^16^ Kooistra et al.^16^ and Cauchois et al.^6^ also investigated whether *anakinra* was effective at reducing clinical signs of hyperinflammation in critically ill COVID‐19 patients. In this study, we found significantly decreased mortality after patients received *anakinra* therapy, but no increase in bacterial/fungal co‐infection risk. Furthermore, there are no reports investigating the effect of *anakinra* on the treatment of fungal co‐infection in COVID‐19 patients.

Inhibitors of IL‐6 or its receptor have been successful in treating other cytokine storm syndromes, or CRS secondary to CAR‐T cell therapies.^5^, ^17^, ^18^, ^19^ Several drugs are available, including IL‐6 receptor inhibitors (tocilizumab and *sarilumab*) and IL‐6 inhibitors (*siltuximab* and *sirukumab*). It is noteworthy that since March 2020 tocilizumab has been formally included in the National Health Commission of China's COVID‐19 diagnosis and treatment program (7th edition): ‘Tocilizumab can be used in patients with extensive bilateral lung lesion opacity or in severe or critical patients, who have elevated IL‐6 levels’. More recently, the Infectious Diseases Society of America has published guidelines and recommends that, for patients who have been admitted to hospital with COVID‐19, tocilizumab should be used only in the context of a clinical trial, due to a ‘knowledge gap’.

Some RCTs have reported that tocilizumab was not effective in preventing intubation or death in moderately ill hospitalised patients with COVID‐19.^1^, ^2^, ^5^, ^17^ However, several studies have found that tocilizumab may be a safe and promising therapeutic option for use in combination with standard care to prevent disease progression in hospitalised patients with moderate COVID‐19 and hyperinflammation.^7^, ^11^, ^12^, ^13^, ^14^, ^18^, ^19^, ^20^, ^21^, ^22^, ^23^, ^24^ Only one article was found to show that overall clinical improvement and mortality in patients with severe COVID‐19 were not significantly different between *sarilumab* and standard care options.^8^

To the best of our knowledge, this is the only systematic review and meta‐analysis conducted to investigate the efficacy and secondary infection risk of immunosuppressants treatment in COVID‐19 patients. In our meta‐analysis, we also found that tocilizumab significantly decreased mortality in COVID‐19 patients without any increased risk of secondary infection. In addition, we found similar results in several subgroups. However, we found that tocilizumab therapy significantly increased the risk of fungal co‐infection in COVID‐19 patients. Therefore, our data suggest that clinicians should be aware of antifungal therapy when COVID‐19 patients are receiving tocilizumab therapy.

There are several limitations to our current study, which needs to be addressed. Firstly, only 33 studies were included, and the relatively small total sample size had limited power for the exploration of real associations. Secondly, subgroup analyses involved relatively small groups, which may not impart sufficient statistical power to explore real associations and are more likely to reveal greater beneficial effects than large‐scale trials. Thirdly, every doctor has a different treatment for clinical diagnostic and treatment algorithms, which would allow for adjustment by other factors.^3^, ^25^, ^26^, ^27^ In addition, the inclusion of zero‐event trials can sometimes decrease the effect size estimate and narrow the CIs.

## 5 CONCLUSION

Overall, immunosuppressants significantly decreased mortality in COVID‐19 patients without any increased risk of secondary infection. Our analyses of tocilizumab therapy showed that there was a significantly increased risk of fungal co‐infections.

## CONFLICT OF INTEREST

The authors declare no conflict of interest in the work.

## ETHICS STATEMENT

All analyses were based on previous published studies; thus, no ethical approval and patient consent were required.

## AUTHOR CONTRIBUTIONS

Jingwen Peng, Meihua H. Fu and Huan Mei conceived and designed the experiments. Jingwen Peng, Hailin L. Zheng and Huan Mei performed publication searches and selection. Jingwen Peng, Qiong Wang and Hailin L. Zheng analysed the data. Jingwen Peng, Guanzhao Liang and Meihua H. Fu prepared the figures. Jingwen Peng and Xiaodong She contributed materials/analysis tools. Jingwen Peng and Weida Liu wrote and revised the study. All authors reviewed the manuscript.

## AUTHORSHIP

All named authors meet the International Committee of Medical Journal Editors (ICMJE) criteria for authorship for this article, take responsibility for the integrity of the work as a whole and have given their approval for this version to be published.

## PERMISSION TO REPRODUCE MATERIAL FROM OTHER SOURCES

We did not reproduce material from other sources.

## Data Availability

The data that support the findings of this study are available from the corresponding author upon reasonable request.
